# Managing Health Concerns Related to Post-Industrial Sites Redevelopment: A Warsaw, Poland Case Study

**DOI:** 10.3390/ijerph20146362

**Published:** 2023-07-14

**Authors:** Agnieszka Zwirowicz-Rutkowska, Joanna Nowak Da Costa, Andrzej Muczyński

**Affiliations:** 1 Faculty of Civil Engineering and Geodesy, Military University of Technology in Warsaw, Kaliskiego 2, 00-908 Warsaw, Poland; joanna.nowakdc@wat.edu.pl; 2 Faculty of Geoengineering, University of Warmia and Mazury in Olsztyn, Prawocheńskiego 15, 10-720 Olsztyn, Poland; amucz@uwm.edu.pl

**Keywords:** health risk, social awareness, information management, urban redevelopment, post-industrial contamination

## Abstract

An important issue in the redevelopment of post-industrial sites, e.g., into housing, is the resolution of contaminated land issues, including health risks and environmental protection. The purpose of this article is to examine awareness of this aspect from the perspective of city or such site residents, using Warsaw as an example. Using a survey-style form data collection technique, a total of 55 fully completed survey questionnaires were collected and analysed using cross-tabulation. Furthermore, a desk research methodology was used to study the availability of sources on industrial areas and their transformation from the perspective of different stakeholder groups involved in the development of such areas in Poland. Similarly, information management was assessed from the viewpoint of information communities participating or being affected by redevelopment processes. The survey results evidenced that respondents are aware that post-industrial sites may be contaminated, but do not associate the possibility of potential health risks when residing on or near such sites. The analysis of the management of information on the reurbanisation of post-industrial sites in urban areas in Poland, including the availability of data on location and contamination, revealed a problematic data flow between central and local level authorities and mootable consistency of legal acts. Public awareness of negative phenomena, such as contamination and the consequent health risks associated with dwelling in such sites, can positively influence, as an input to monitoring and enforcement, the actions taken by other stakeholders in the clean-up processes of contaminated sites and force improvements in the management of such information. The flow of information, the activities forming the information function, and the decision-making process can be improved by technologies, such as spatial information systems and their infrastructures, by facilitating the integration of data from multiple sources and consequently enabling the analysis to be extended to include further relevant data.

## 1. Introduction

The phenomenon of industries abandoning their existing locations occurs worldwide at different time periods and with varying degrees of intensity. A post-industrial site (often referred to as brownfield) is a degraded, disused or under-used site that was originally intended for economic activities that have, however, already terminated. In a broader sense, brownfield sites include areas degraded by industrial activities, e.g., mining or quarrying, manufacturing, dumping of industrial waste, soil or water contamination, etc. These are areas where industrial production took place directly and areas with ancillary functions within the plants, e.g., administrative, storage or transportation. 

The redevelopment of post-industrial sites includes technological, social and environmental measures. It involves both the elimination of the harmful effects of the contamination present at the site (i.e., remediation), and the recreation of a sustainable ecosystem. Such biological activation of the area, i.e., rehabilitation, is also part of the revitalisation. The latter encompasses the process of redevelopment of an area, including the cleaning, redevelopment and upgrading of the existing land cover, which will return brownfield to a state that allows it to perform its useful urban functions. 

Redeveloping brownfield sites and facilities is a widespread problem [1,2,3,4,5,6] and involves various technological, social and environmental activities [5,7]. Because of the significant impact of deindustrialisation on city functioning, redevelopment of brownfield sites and facilities has become a major challenge for municipal and regional authorities and governments of industrialised countries due to its scale, environmental and social risks [8,9].

The redevelopment of post-industrial sites is now a key issue for those concerned with spatial planning, sustainable development, environmental protection and land and property ownership, and involves cooperation between different stakeholders [10,11], including public participation [12,13]. 

The possibilities of converting post-industrial buildings for residential use are quite complicated due to the relatively high technical difficulties and high costs of redevelopment [14]. Problems that have to be faced when planning the revitalisation of abandoned post-industrial sites include the need to improve the quality of the environment, create a new vision for the urban area, create new activities and services in the vicinity, as well as to cope with numerous architectural and ownership barriers and restrictions related to conservation requirements. Post-industrial development is also characterised by specific physical features—size, form and design [8]. At the urban scale, restructuring solutions should implement the principles of a sustainable environment, which is one element of a broadly integrated strategy that takes into account the local, regional and global impact of transformed areas on soil, air, vegetation, fauna and human population [15,16,17].

The conversion of such land for residential purposes should meet the basic living needs of future residents, which are related to the concept of quality of life [18]. Objective quality of life defines the conditions of human life, while subjective quality of life is an assessment of the degree to which needs are satisfied [19]. Among the indicators of objective quality of life, the following are most often mentioned: health care, safety, the state of the natural environment, the standard of living of the inhabitants, and accessibility to services and public facilities [20,21]. Therefore, an important issue in the transformation of such sites is the resolution of contaminated land issues, including but not limited to health risks.

The topic of contaminated sites following the cessation of industrial activities or waste disposal or mineral extraction in the context of health risks, including morbidity and mortality of local, i.e., exposed populations, has been addressed by researchers worldwide [22,23,24,25,26,27,28]. However, there are very few studies on the extent to which the remediation of contaminated sites affects (reduces) health risks to new and existing populations, reporting on health outcomes in humans before and after remediation or redevelopment of contaminated sites. A recent effort to search for such data and studies in [29] ended up finding only 16 items. Virtually each of them dealt with different contaminations, and each study was designed differently; hence common conclusions can hardly be drawn. However, there are studies that show changes in lead and chromium concentrations in children’s blood and urine after soil remediation and, in some cases, after public health campaigns to reduce exposure [30].

Land contamination, particularly caused by industrial contamination, is also a target of interest for various organisations, including the WHO, who are encouraging efforts towards environmental justice to reduce environmental exposure and associated health risks [31,32,33]. The importance of understanding and quantifying the risks associated with the hazards, including in the context of contaminated land management and its redevelopment, is underlined by [34,35]. There are postulates of detailed identification of hazardous substances present in degraded land and analysis of their potential effect on people, as well as standardisation of good practices in the decommissioning of industrial sites process [36,37,38].

The aim of the article is twofold. Firstly, to analyse the awareness of health risks from the perspective of city dwellers, current or future residents of settlements on post-industrial sites in the example of the city of Warsaw. The second objective is to analyse the availability of data sources on such sites and their reurbanisation in Poland and to assess this model of information management from the perspective of the entire information community participating in revitalisation processes.

## 2. Methods

### 2.1. Study Area

Warsaw used to be a city with a crucial and strongly developed industrial function. The process of industrialisation intensified after the Second World War, while the deindustrialisation of Warsaw, one can assume, took place from the late 1970s onwards. There was a comprehensive restructuring of industry and structural transformation. During this deindustrialisation, decommissioned enterprises created degraded areas, which are currently under consideration or have recently been redeveloped in many directions, not only economically, including residential or commercial roles, but also culturally.

**Figure 1:**
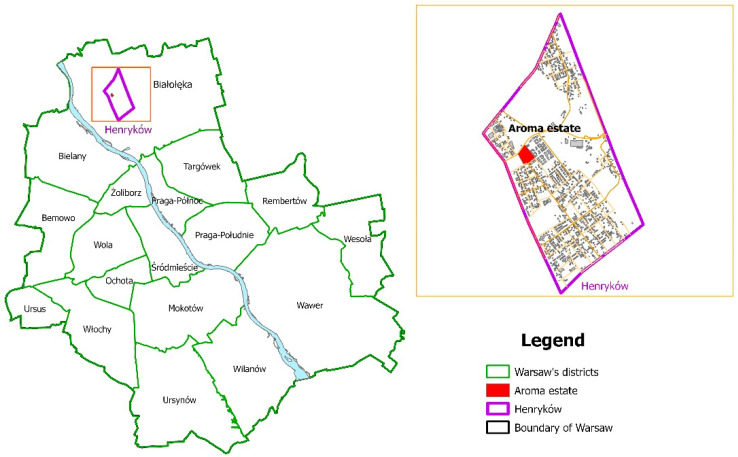
Map of Warsaw districts with the Henryków sub-district and the Aroma estate marked.

We chose the industrial complex in Henryków, originally a small town in the northeast, not far from the then borders of Warsaw, as an example use case. Now, it is a part of the Białołęka district of Warsaw (Figure 1). A plant-producing baker’s yeast was established in 1902 by the entrepreneur Henryk Bienenthal, after whom the place took its later name of Henryków. Until the outbreak of the Second World War, the plant operated very dynamically, while after the war, there was a break of about 10 years, and it was not until 1955 that the production of fragrance synthetics was launched there. The Pollena-Aroma Fragrance Factory operated on the site until 2012, when it moved to its headquarters in Nowy Dwór Mazowiecki, a town 25 km to the southeast of Henryków [39].

After the move, the complex was completely inaccessible, and at the turn of 2016/2017, Pollena sold the three-hectare plot of land in Henryków. It was bought by the Finnish investor YIT for residential development. Eight historic buildings were preserved at the time, four of which survive almost unchanged, including the first and only surviving yeast distillery in and around Warsaw. In 2019, some of the buildings were entered into the register of historical monuments because of their testimony to the links between residential and industrial architecture, artistic preferences and the prestige enjoyed by the distillery near Warsaw. Developer YIT demolished the uninteresting, post-war buildings and began construction of residential buildings and renovation of the 100-year-old structures. 

During the works, the topsoil under some buildings was found to be contaminated with C12-C35 hydrocarbons and oil fraction components, barium (Ba), zinc (Zn), copper (Cu), lead (Pb), ethylbenzene, benzene, tetrachloroethene, dichloroethene or trichloroethene. The presence of different contaminants results in different remediation methods, but each is a relatively long-term process, i.e., between 1.5 and 3.5 years. As a result, one such process is still in progress, even though the estate is already operational. 

It is worth mentioning that in the case of the soil remediation under the site’s main building, the developer requested to demolish it and then rebuild it from scratch. The city conservation office refused, stating that the historic walls were not eligible for complete demolition. Consequently, the whole building was lifted and returned to its previous location once the site was remediated. The developer has also been concerned about protecting the greenery. It makes up 40 per cent of the Aroma estate (as it is now called), including a priceless old-growth forest of twenty-some old trees, lime, ash and maple, among others [40].

### 2.2. Survey

The survey was addressed to residents of Warsaw and aimed to obtain information on public awareness of the health risks that may be associated with living on or close to former industrial sites. We also investigated awareness of the existence of and access to information on historical land contamination. The environmental awareness aspect of current and future generations was also surveyed.

The questions were formulated independently against each other, and their number was reduced to the minimum to keep it less tedious for respondents and reach a high number of participants. The survey here in question reported entirely in Supplementary Material 1, consisted of 12 questions structured according to the methodology present in [41]. Most of them are single-answer multiple choice question types. Two questions, namely the fifth (Q5) and nine (Q9), are the multiple-answer multiple choice questions. They included a predetermined list of options, and allowed respondents to check off all the choices that apply to them. If none of the answer options fulfilled their expectations, they could choose an “other” answer option listed at the end of all choices, and fill in their answer. 

One survey question is both a multiple-answer multiple choice and a ranking question (namely Q9). It asks respondents to order answer choices by way of preference. This type of question requests respondents’ familiarity with each answer option. Therefore, we used it only once in our survey, and for a subject that is familiar to city inhabitants.

The survey contained nine substantive questions, followed by three demographic questions (on age, gender and education level) to sociologically characterise the responses. The answers were collected using a paper hardcopy version of the survey as we were pooling pedestrians in the different parts of Warsaw. The collected data were statistically analysed using the cross-tabulation method. 

### 2.3. Analysis of Data Sources on Post-Industrial Sites

A selection of potential sources of knowledge on post-industrial sites was made based on legislation, websites of public administrations and research institutes, government agencies, archival cartographic material, hobbyist studies, Internet postings, newspaper articles and non-governmental urban and tourism portals.

The analysis of the data sources was based on seven criteria, which are used as metadata elements for spatial data resources and constitute characteristics of the data [42,43], as well as on the paradigm of open data [44,45]. The considered criteria are as follows (Table 1): Online availability (where possible options are Yes/No), Registration (Yes/No), Access (public/public administration/others), Type (analogue/digital), Form (map/document/register), Organisation responsible for dataset (central public administration, local public administration, regional public administration, public service institution, company distributor, research institute, volunteer), Area of data coverage (whole country/selected regions/selected districts/selected municipalities/selected parts of the municipality/selected parts of the district), Case study area (i.e., post-industrial area of Henryków Pollena Aroma) inclusion (Yes/No).

### 2.4. Assessment of Information Management with Respect to the Implementation of Tasks and Solving Problems Related to the Redevelopment of Post-Industrial Sites

Information management includes both a set of activities forming the information function of an organisation, i.e., information acquisition, information processing, information integration, and the activities within the framework of planes (technological, organisational, legal) affecting the realisation of this function [46,47,48]. In this paper, the information management issue is related to the information community consisting of e-government, citizens and other participants in post-industrial urban areas transformations, including research institutes, government agencies or some professionals like planners and urban planners, real estate professionals, investors and developers. The information function is related to the ability of the information community to carry out tasks and solve issues related to the transformation of post-industrial sites, using the topic of health safety as an example.

**Table 1:** Results of the analysis of potential sources of knowledge on post-industrial or degraded sites.

No.	Resource	Online Availability	Registration	Access	Type	Form	Responsible Organisation	Data Coverage Extent	Case Study Area Inclusion
1	Register of historical land surface pollution	No for the public [49] Yes, for public administration	Yes	public	digital	Register (excel file)	central public administration	whole country	Yes
2	Register of environmental damage and imminent threats of environmental damage	No for the public [50] Yes, for public administration	Yes	public	digital	Register (excel file)	central public administration	whole country	Yes
3	Revitalisation statistics at the municipality level	Yes [51]	No	public	digital	document	public service institution	whole country	Yes (county scale only)
4	Map of degraded and elevated natural hazard areas at a scale of 1:10,000	Yes [52]	No	public	digital	map	public service institution	selected parts of municipalities	No
5	Map of potential land contamination	Yes [53]	No	public	digital	Online map	volunteer	selected district of Warsaw	No
6	City of Warsaw—Spatial Information System	Yes [54]	No	public	digital	Online map	local public administration	city of Warsaw	Yes
7	Map portal of the City of Warsaw, Office of Architecture and Spatial Planning	Yes [55]	No	public	digital	Maps and documents	local public administration	city of Warsaw	Yes
8	Geochemical atlas of Warsaw and surroundings 1:100,000	Yes [56]	No	public	digital	map	public service institution	city of Warsaw	Yes (sub-district scale only)
9	Cartographic geochemical studies in Poland	Yes [57]	No	public	digital	Maps and documents	public service institution	city of Warsaw	Yes
10	Geoportal of the capital city of Warsaw	Yes [58]	No	public	digital	Maps and documents	local public administration	city of Warsaw	Yes
11	Polish National Geoportal	Yes [59]	No	public	digital	Maps and documents	central public administration	city of Warsaw	Yes
12	Local Revitalisation Programme of the City of Warsaw	Yes [60]	No	public	digital	Maps and documents	local public administration	selected district of Warsaw	No
13	Warsaw’s Spatial Development Conditions and Directions Study	Yes [61]	No	public	digital	Maps and documents	local public administration	city of Warsaw	Yes (sub-district scale only)
14	Ecophysiographic Atlas of Warsaw	Yes [62]	No	public	digital	Maps and documents	local public administration	city of Warsaw	Yes (sub-district scale only)
15	Integrated Revitalisation Programme	Yes [63]	No	public	digital	documents	local public administration	selected district of Warsaw	No
16	Białołęka local development plans (Białołęka—miejscowe plany zagospodarowania przestrzennego)	Yes [64]	No	public	digital	Maps and documents	local public administration	selected district of Warsaw	No
17	Environmental Protection Programme for the Mazowieckie Voivodeship until 2030	Yes [65]	No	public	digital	documents	local public administration	selected district of Warsaw	No
18	POIs in the Industry category in Warsaw and surroundings	Yes [66]	No	public	digital	Online map	company distributor	selected district of Warsaw	Yes (but no items)

The evaluation of information management in the implementation of tasks and the resolution of problems related to the transformation of contaminated sites, using the example of health risks, is understood in this publication as operational information management. The analysis of such management is examined from the following three perspectives.

The organisational perspective includes the distribution of information management competencies between actors, stakeholders and other participants in post-industrial site redevelopment activities with specific tasks and roles on the one hand, and the organisation of information processes on the other. This study takes into account organisational interoperability, i.e., information on access to information resources, standardisation and standardisation of procedures from ensuring correct collaboration of the geo-information community (potential options: low/medium/high). We consider the distribution of competencies and roles (potential options: Yes/No/Partially) and the existence of procedures for organising information processes (Yes/No/Partially).

The technological perspective refers to the occurrence of ICT systems, databases, spatial databases, information and geo-information infrastructures that support the informational function, but also the level of usability of the technology to perform the activities that constitute this function and the level of interoperability between technological solutions used to perform such informational function. The occurrence of technology is assessed as: low—if no or little technology is implemented to single informative tasks or solutions; medium—if some technology solutions, of relatively high dispersion are implemented to a single or group of informative tasks; and high—if there are many technology solutions, implementation to group or all of informative tasks, some hub or main access to technological solution. The level of usability of this technology can be assessed as low in case it does not satisfy information needs in the implementation of selected activities, medium if it satisfies information needs in selected aspects of activities undertaken, or high provided it satisfies needs in most or all activities. In turn, the level of interoperability can take the following values: low—no use of agreed or recognised standards and norms recognised in good practice by international organisations; medium—there is evidence of use of agreed or recognised standards and norms recognised in good practice by international organisations in selected aspects of the information function, or high if international organisations in selected aspects of the information function recognise common use of agreed or recognised standards or good practice norms.

The legal perspective is represented by the formal and legal regulation of the information and communication function of the post-industrial land transformation community. We examine the existence of formal and legal regulations. It is regarded as ‘No’ if there is no policy and regulations on information management among information community; ‘Partially’—if there is some policy for selected aspects of tasks in the implementation of brownfields transformation, and not an overall government-wide policy on information management between different participants of information community; and finally answer is ‘Yes’ provided policy and regulations for tasks in the implementation of brownfields transformation are very complex I government-wide policy on information management between different participants of information community. While the level of consistency between formal and legal regulations is evaluated as ‘low,’ ‘medium’, or ‘high’.

The data for the assessment of information management in the field of task implementation and problem-solving in the field of post-industrial urban area redevelopment were obtained on the basis of in-depth literature reviews, empirical research results available in the form of reports and qualitative and quantitative research relating to the use case presented in Section 2.1, Section 2.2 and Section 2.3 and concern a summative approach taking into account different stakeholder groups.

## 3. Results

### 3.1. Level of Public Awareness

The survey made it possible to assess people’s awareness of some risks of living on or near post-industrial sites, using the example of Poland’s capital city. It should be pointed out that this survey has been carried out both in a local context where people have been already living in a residential area created on the post-industrial land and in various groups of people not belonging to such society or at least we are not aware of it. 

Responses were collected using a paper version of the survey as we polled pedestrians in different parts of Warsaw. A total of 55 respondents took part in the survey. The basic demographic statistics are as follows. The gender distribution among respondents is practically even, while in terms of age, younger people predominate. People under 20 years of age account for 58% of all respondents, and people under 40 years of age account for 74% of all respondents. The majority of respondents had a university degree (80%), of which 53% had a bachelor’s degree.

**Figure 2:**
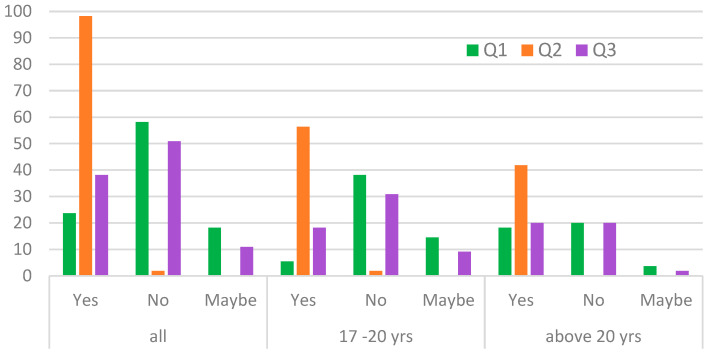
Group I questions: Willingness to live in post-industrial areas and awareness of potential pollution or health threats (in percentage, by age).

The first group of questions was related to exploring the aspect of awareness of the possibility of living in post-industrial areas and its potential threats or risks. We asked respondents whether they would consider buying or renting a flat in a post-industrial area or in the immediate vicinity of areas still used by industry, e.g., steelworks, combined heat and power plants, manufacturing, chemical plants, factory (Q1). The majority of respondents are not interested in living in such areas, as Figure 2 illustrates. 

The next question was about awareness of potential pollution in such areas (Q2). Most of the respondents are aware that there may be contamination of the land on and in the immediate vicinity of post-industrial areas, e.g., heavy metals, organic compounds (Figure 2 ). Moreover, the majority of respondents, independent of their age, do not associate the fact of living in such areas with potential impacts (i.e., risks, consequences) on the residents’ health (Q3, Figure 2). Such responses are a kind of explanation for such a large number of negative answers to the first question (Q1), despite the fact that the survey was preceded by an introduction lasting several minutes, during which potential survey respondents were acquainted with the phenomenon of transforming post-industrial areas into residential areas and briefly presented the obligations of the owner of the transformed plots in the context of adjusting the area to the goal of the health of future residents of these areas.

**Figure 3:**
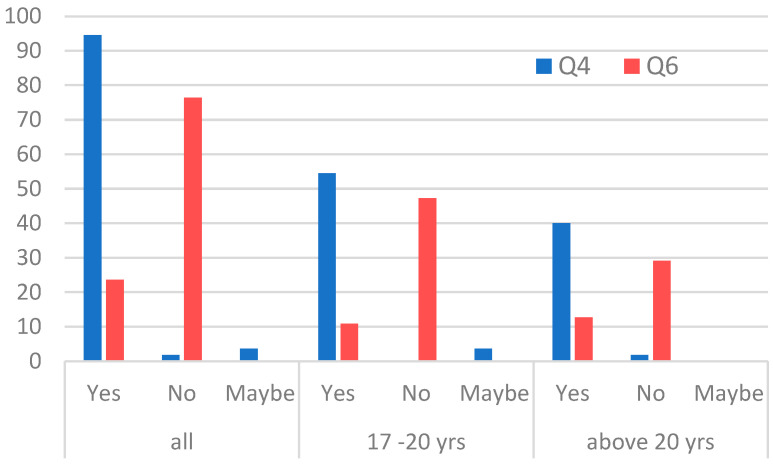
Group II questions: Respondents’ awareness of and access to information on potential historical land contamination and other risks or consequences associated with inhabiting post-industrial areas or directly adjacent to such areas (in percentage, by age).

**Figure 4:**
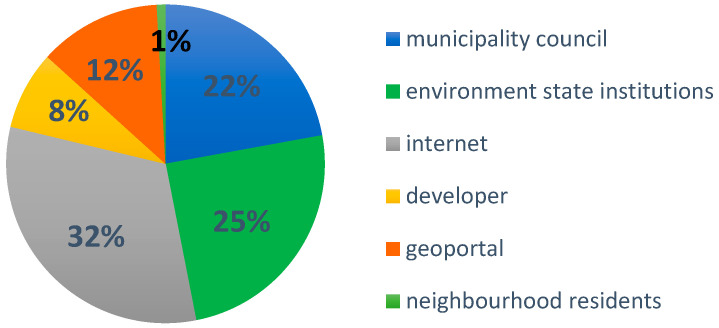
Respondents’ points of reference for information on potential pollution.

The section of the survey on awareness and access to information on historic land contamination consisted of three questions. In the first question (of group II), we asked participants whether they would be interested in information on potential historical land contamination and other risks (consequences) associated with residing on brownfield sites or immediately adjacent to sites still used by industry (Q4, Figure 3). Here, as expected, it was confirmed that the majority of respondents were interested in such information. In another question from this group of questions, we checked whether our respondents were aware of the existence of the State Register of Historical Land Surface Pollution (Q6, Figure 3). It turned out that the majority is not aware of its existence, although the information contained in the Register is available to the potential future resident of brownfield sites and every citizen. The question arises, how to look for such information if not in the Register? The answer to the question (Q5) showed where our respondents would look for such information. The most frequently mentioned reference points or institutions turned out to be, in addition to the Internet search engine, the municipal council and the state institutions dealing with environmental protection (Figure 4).

**Figure 5:**
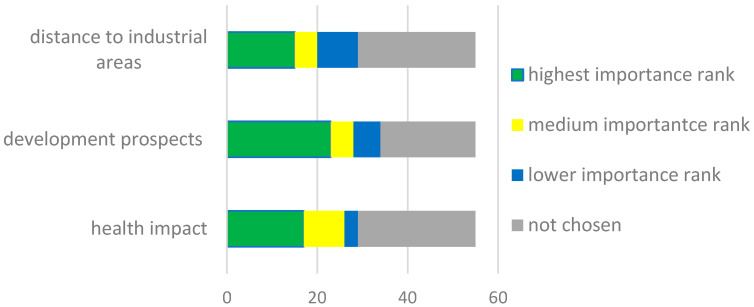
Respondents’ ranking regarding decisive factors while selecting an apartment.

Respondents were also asked to indicate eight factors out of a predetermined list of options that would be decisive in selecting an apartment, and rank their importance. The health impact of existing or historical pollutants in the area (e.g., air, water, ground) or noise emitted into the environment was chosen by 53% of respondents as one of the eight decisive factors (Figure 5). For 47% of survey participants, the health impact was ranked as the highest or medium importance (among these eight indicated factors).

The survey showed the following factors: development prospects and distance to industry sites are important to 62% and 53% of respondents, respectively, of which 51% and 36% of survey participants gave them a high or medium priority among the eight selected factors (Figure 5).

**Figure 6:**
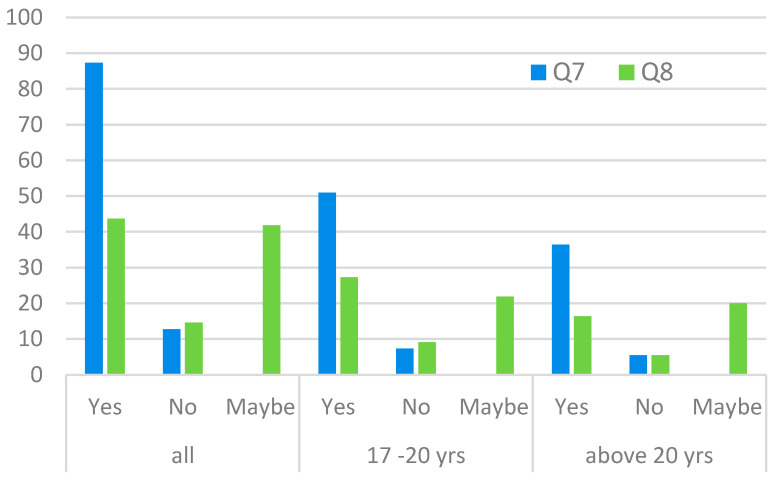
Group III questions: Respondents’ concern for environmental protection and minimising the ecological footprint (in percentage, by age).

The last group of questions concerned the aspect of environmental awareness for temporary and future generations. We wondered whether a future/current resident of a post-industrial area might be interested in whether the Developer has taken care of the environment (Q7) and whether the ecological footprint, in general, is of great importance to the respondent in question (Q8). It turned out that the majority of residents, independently of their age group, would like to do their part to protect the Earth’s environment (Figure 6) by choosing a Developer who cares about the environment, but only half are interested (or aware) of minimising the ecological footprint.

### 3.2. Information Availability

Almost 20 data sources on industrial, degraded and post-industrial sites undergoing redevelopment in the Warsaw area were collected and analysed. Public access restrictions, access forms, geographical coverage or source lineage were assessed. 

The results are presented in Table 1. Data on brownfield sites and their potential pollution can be found in various studies by a number of actors. The first source of data is the repositories of the General Directorate of Environmental Protection (Table 1, no. 1–2). Another source of information is the local and regional administrations. In the example under consideration, these are the resources of the Warsaw City Hall (nos. 6–7, 10–16) and the studies of the Marshal’s Office of the Mazowieckie Voivodeship (no. 17). The subject is also taken up by the Geological Survey (nos. 4, 8–9 in Table 1). Data on the post-industrial sites are also integrated into the National geoportal run by the State geodetic and cartographic service. Another data provider is the statistical service (no. 3 in Table 1) and citizen studies (no. 5), or even commercial companies (no. 18). The analysed data sources varied in form (Table 1, Form column), and 12 of them (i.e., 66%) have the study case area covered.

### 3.3. Assessment of Information Management on Post-Industrial Sites’ Redevelopment

Table 2 shows the results of the assessment of information management in terms of tasks and problem-solving related to the transformation of post-industrial sites in Poland from the perspectives of different stakeholder groups. From an organisational perspective, both the division into tasks and roles and the existence of appropriate procedures for organising information processes were assessed as partial. There is a division of responsibilities and organisation of selected information processes within individual entities and institutions related to the selected processes of brownfield conversion. From the environmental perspective, the subject of the change of use of post-industrial sites to residential areas is related, among other things, to land surface pollution, including historical pollution. The entities conducting spatial policy tasks focus on the issues related to the reclamation and revitalisation of such areas. Tasks attributed to the Geological Survey, which conducts statistics, as well as to central and local public administration and citizens (residents), can also be outlined. Information on access to information resources, standardisation and unification of procedures from ensuring proper cooperation of geoinformation community expressed through organisational interoperability was assessed as ‘low.’

Mapping studies, databases and registers maintained by individual entities using information and communication technology can be identified (Table 1); however, they are characterised by a high degree of dispersion. There are also a very high number of studies in analogue form, so the assessment of the aspect of the existence of systems, databases and infrastructures (within the technological perspective) is ‘partial.’ From the usability perspective of these technological solutions, it can be pointed out that many of them are local or regional in nature. Resources cover data piecemeal or over selected time periods. Therefore, the level of this was assessed as ‘low.’ The technological interoperability of these solutions was assessed as low due to the lack of agreed standards for data exchange and access to resources.

With regard to the incidence of formal and legal regulation of activities related to the information function, it was estimated to be ‘partial,’ as there is a lack of an overall government-wide policy on information management between different participants of the information community, for example in terms of accessibility to data by citizens. The level of consistency of the legislation was assessed as ‘medium’ since the issue of post-industrial areas’ transformation is anchored in several Polish laws, i.e., Environmental Protection Law, Act on the Protection of Agricultural and Forestry Land, Geological and Mining Law, Act on Planning and Spatial Development. The analysis of legal acts shows that in the design and implementation of revitalisation projects, a multitude of regulations should be used. Unfortunately, this multiplicity of legal norms, the lack of cohesion of individual provisions, as well as the lack of regulation of certain issues and, in admittedly few cases, the failure of the administration to implement the provisions of the law (e.g., the lack of local spatial development plans in communes) complicate the procedures. The complexity of these procedures is further caused by the need to obtain opinions and agreements of various institutions and organisations.

## 4. Discussion

A poll survey was carried out to verify people’s awareness of the risks of residing on or near post-industrial sites, using the example of Poland’s capital city. The results confirmed that respondents are aware that post-industrial sites may be contaminated, but do not associate the possibility of potential health risks with living on or near such sites. The predominant criterion for the decision to buy or rent a flat (Q9) is the price per square metre. The criterion of distance from factories, chemical plants, thermal power plants and the criterion of the health impact of existing or historical pollutants in the area (e.g., air, water, ground) or noise emitted into the environment were important but not decisive criteria. The other criteria for Q9 are commonly used in real estate appraisal and trade (e.g., [67,68,69,70,71]).

An additional issue was to verify whether respondents were interested in environmental issues in the case of development projects, especially on brownfield sites. Site contamination may include, in addition to soil contamination, contamination of post-industrial buildings, and hazardous materials (e.g., asbestos). The application of appropriate procedures by the developer in the processes of removing contaminants, securing the storage and disposal of materials that threaten the safety of people and pose a threat to the environment, making the entire development project as ecologically optimised as possible can be a manifestation of its commitment and interest in environmental protection. This commitment, as the results of the survey have shown, is expected and socially desirable.

It should also be noted that respondents confirmed their desire to access information on post-industrial sites and the possible environmental and health risks associated with the conversion of these sites to residential use. On the one hand, they indicated public administrations as a source of information; on the other hand, the internet. 

The analysis of data sources on industrial degraded, and post-industrial sites undergoing redevelopment for the study area made it possible to verify the accessibility of information for a wide range of users. These sources are widely available on the internet. Many of them are accessible through geoportals. However, in the surveys, a small percentage of respondents identified map portals as a source of information. The available sources of information are characterised by different forms and spatial coverage, but also by a high degree of dispersion due to the fact that they are made available by different public administration bodies, both central and local, in relation to the assignments they carry out. The availability of data created by volunteers, i.e., citizen science data, should be highlighted as it plays an important role in informing the public about potential health risk sites, but also provide a source of knowledge for public administrations to initiate the relevant procedures for investigating such a site and then remediating it.

The assessment of information management with regard to tasks and problem-solving related to the redevelopment of brownfield sites in Poland from the perspective of different stakeholder groups revealed in a complex way problem domains in the implementation of the information function within the framework of technological, organisational and legal dimensions and confirmed former studies, e.g., [14,16,72,73]. These also included the issue of the health safety of residents, which was perceived from a spatial planning perspective [36].

In the context of the progressive development of e-government in Poland, which includes a number of stakeholders also involved in tasks related to the transformation of brownfield sites, as well as the national programme for the development of open data, there is an opportunity to increase the level of efficiency of information management in the framework analysed in this article. The Regulation on Interoperability of Public Administration ICT Systems and the Spatial Information Infrastructure Act [74,75], which is a direct transposition of the INSPIRE Directive [76], as well as open data [77], are of great importance for this process. In addition, measures such as developed databases and geographic information systems can play an important role in coordinating activities and supporting decision-making in the management of brownfield information, examples of which can be found in Poland, but also in other countries, e.g., in [78,79].

## 5. Conclusions

The redevelopment of brownfield sites, including for use as residential accommodation, is a multifaceted and interdisciplinary issue. Such conversions involve addressing a number of problem domains, including those related to health and environmental risks, but not limited to. Consequently, it becomes necessary to analyse, and even acquire (measure) in advance, a wide variety of data, and to take decisions and actions that involve many different stakeholders. This article addresses the issue of public awareness of the possible risks of inhabiting such areas. The reference point was the case of the city of Warsaw, the capital of Poland.

The research included a survey of residents, and an analysis of data sources and their management. In addition to providing input from residents, the survey can be an instrument used to stimulate public awareness of the possible effects and consequences of living on post-industrial sites with pollution for a long time and be used at the public consultation stage in various tasks related to the transformation of post-industrial sites. The data analysis took into account criteria relevant from the perspective of the universality of access to data and can contribute to the development possibilities of e-government in Poland. Whereas the analysis of information management between different stakeholders of processes related to brownfields transformation allowed us to identify problem areas related to access to data, their quality and cooperation in solving problem domains and a comprehensive approach to issues with the use of ICT and geospatial technology solutions.

This study contributes to (a) the consideration of the social dimension of brownfield redevelopment and the provision of quality of life in such areas, as well as to (b) the perception of health security as an important factor of well-being, and to (c) the screening of public awareness in this regard. From the perspective of the problem-solving processes involved in transforming such areas, this research contributes to the issue of evaluating data sources and factors that improve information management, which ultimately translates into the quality of decision-making processes.

The results of the research, both survey questionnaire and desk research, can be used by bodies and stakeholders involved in the transformation processes to formulate an information campaign, popularising the topic of transformation of post-industrial areas into residential areas, taking into account the risk of the impact of contaminants on health. Equally important is a bottom-up initiative, popularising the results through social networking sites, which can help to stimulate public awareness of the possible effects and consequences of living for a long time in post-industrial areas with contaminated sites. Here, again, it is worth noting the citizen science service, which, on the basis of old descriptions, books and historical accounts, indicates potential land contamination for Warsaw’s Wola district, and was intended as a clue or even trigger for administrative bodies in taking measures to identify contamination.

In the context of the legal regulations in Poland, it is particularly crucial to inform investors that already at the stage of the construction project, it is necessary to assess the degree of contamination of the ground and to select the method of land treatment on the basis of soil and groundwater contamination studies carried out. It is also important to strengthen the monitoring procedures for the actions taken and the pollution removed by developers. The inclusion of information on the carbon footprint by the developers in the implemented investment, and in the advertising prospectuses of the settlements, as proposed in this article, can contribute to the popularisation and development of environmental awareness in society.

It is proposed to integrate data on post-industrial sites within the spatial information infrastructure in Poland, according to the rules of technological interoperability. On the technological side of infrastructure operation, an important advantage is the ability to integrate resources (data and information) in one place, to analyse and visualise them together. As a result, its information value is increased. From the perspective of public administration bodies, there are already legal solutions, towards owning and managing resources using ICT systems. Many of the bodies that could provide these data in the context of post-industrial land transformation, and which were referred to in the analysis of sources (see Table 1), are either central or local level administrations that maintain their resources to fulfil the INSPIRE and the National Spatial Data Infrastructure projects. Intensified action (e.g., additional rules) is still needed to integrate data information from these institutions.

From the perspective of decision-making based on spatial analyses, mentioned data integration makes it possible to carry out more in-depth analyses, as they are based on more criteria.

For the resident of the municipality, but also for every citizen, it is important to properly manage information about land contamination risks. Universal access to a web-based system of historical land contamination records and making such information public, preferably in the form of another information layer in a municipal GIS, would increase access to information, the ability to monitor, for example, dishonesty or shortcomings on the part of land owners, users or developers, as it is their responsibility to investigate the condition of the land and its remediation. In addition, the data can be integrated into citizen science services, thus increasing the level of organisational interoperability.

The analysis presented in this study focuses on the problem area of health risks of residents that arise in the redevelopment of brownfield sites and covers the chosen direction of transformation, i.e., residential development. The issue of assessing such risks and the information management model in this aspect among the actors involved in the transformation processes are also relevant from the perspective of designing other functions for such sites, e.g., water, recreation or agricultural functions.

## Figures and Tables

**Table 2:** Results of information management assessment of post-industrial sites redevelopment tasks and solutions.

Perspective	Organisational		Technological		Legal	
Aspect	Tasks and roles	partial	Systems, databases, infrastructures	partial	Formal and legal regulations	partial
	Organisation of information processes	partial	Technology usability	low		
	Organisational interoperability	low	Technological interoperability	low	Consistency of formal and legal regulations	medium

## Data Availability

Data used in this study were derived from public and public administration websites listed in the References section.

## Supplementary Materials

The following supporting information can be downloaded at: https://www.mdpi.com/article/10.3390/ijerph20146362/s1.
