# Supplementary material for: Managing Health Concerns Related to Post-Industrial Sites Redevelopment: A Warsaw, Poland Case Study
Source: Int J Environ Res Public Health. 2023 Jul 14;20(14):6362. doi: 10.3390/ijerph20146362 (PMC10379291; doi:10.3390/ijerph20146362)
Supplement: Supplementary file 1 [file ijerph-20-06362-s001.zip › ijerph-2466998-supplementary.pdf]

Managing health concerns related to the redevelopment of post-industrial sites: a Warsaw, Poland, case study

Agnieszka Zwirowicz-Rutkowska, Joanna Nowak Da Costa, Andrzej Muczyński

Supplementary Material 1

The survey was administered in the Polish language. Below we present the survey questions translated to English.

(Q1) Would you consider buying or renting an apartment in a post-industrial area or in the immediate vicinity of an area still used by industry (e.g., steelworks, combined heat and power plant, chemical works, factory, etc.)?

(a) Yes; (b) No; (c) Hard to say

(Q2) Are you aware that soil may be contaminated in post-industrial areas, e.g., heavy metals and organic compounds?

(a) Yes; (b) No

(Q3) Would you associate residing in post-industrial areas or in the immediate vicinity of areas still used by industry with potential impacts (risks, consequences) on your health and loved ones?

(a) Yes; (b) No; (c) Hard to say

(Q4) Would you be interested in information on potential historical land contamination and other risks (consequences) associated with inhabiting post-industrial areas or directly adjacent to areas still used by industry (e.g., smelter, thermal power plant, manufacturing, chemical plants, factory, etc.)?

(a) Yes; (b) No; (c) Hard to say

(Q5) Where would you look for information on potential historical land contamination and other risks (consequences) of living in post-industrial areas or directly adjacent to areas still used by industry (e.g., steelworks, combined heat and power plant, factories, chemical plants, factory, etc.)?

(a) From the developer; (b) From the municipality (city council);

(c) From the state institutions dealing with environmental protection; (d) Internet research;

(e) Municipality/city (geo)information or e-government service; (f) Other (please provide a name). ....

(Q6) Are you aware of the existence of a State Register of historical land surface pollution?

(a) Yes; (b) No

(Q7) If you were buying an apartment in a post-industrial area that required the developer to undertake remediation of contamination, would you be interested to know whether the developer made provisions for the environment if the development was built on the site?

(a) Yes; (b) No

(Q8) Does the ecological footprint of a developer matter a lot to you, e.g., would you choose a developer with a reduced ecological footprint?

(a) Yes; (b) No; (c) Hard to say

(Q9) Indicate eight (8) factors of the following that would be decisive in selecting an apartment. In addition, rank the importance of each factor on a scale of 1–5, where 1—most important and 5—least important of the selected decisive factors:

(a) Distance from place of work (place of study) or family;

(b) Price per square metre;

(c) The spatial development prospects of the area (zoning in the local development plan);

(d) Proximity to nature, parks, recreational areas, and cycle paths;

(e) Accessibility to transport, services, shops, etc.;

(f) Size of the flat (metreage);

(g) Standard of finishing;

(h) Technical condition of the apartment;

(i) Location in relation to the centre of the city;

(j) Age of the building;

(k) Health impact of existing or historical pollutants in the area (e.g., air, water, ground) or noise emitted into the environment, etc.;

(l) Provision of a basement;

(m) Provision of a balcony;

(n) Distance from factories, chemical plants, thermal power plants, steelworks, etc.

(o) Number of rooms;

(p) Closed kitchen (i.e., separate from dining space);

(q) Floor number;

(r) History of the place and neighbourhood.

(Q10). What is your age?

(a) Under 20 l.; (b) 21–30 l.; (c) 31–40 l.; (d) 41–50 l.; (e) 51–60 l.; (f) Over 61 l

(Q11). What is your educational background?

(a) Elementary; (b) Secondary; (c) First degree; (d) Second degree; (e) Above university level

(Q12). What is your gender?

(a) Female; (b) Non-binary; (c) Male; (d) Other
